# Supplementary material for: Display of malaria transmission-blocking antigens on chimeric duck hepatitis B virus-derived virus-like particles produced in Hansenula polymorpha
Source: PLoS One. 2019 Sep 4;14(9):e0221394. doi: 10.1371/journal.pone.0221394 (PMC6726142; doi:10.1371/journal.pone.0221394)
Supplement: S1 Appendix — (DOCX) [file pone.0221394.s001.docx]

**Supplementary material**

of the manuscript entitled

**Display of malaria transmission-blocking antigens on chimeric duck hepatitis B virus-derived virus-like particles produced in *Hansenula polymorpha***

Authors:

David Wetzel, Jo-Anne Chan, Manfred Suckow, Andreas Barbian, Michael Weniger, Volker Jenzelewski, Linda Reiling, Jack S Richards, David A Anderson, Betty Kouskousis, Catherine Palmer, Eric Hanssen, Gerhard Schembecker, Juliane Merz, James G Beeson, Michael Piontek

**Section S1: Additional data on the chimeric Pfs25-dS/dS VLP**

**S1.1 Cross-reactivity of anti-Pfs25 antibodies**

Fig 1 A of the main manuscript shows Western blot analyses on the chimeric Pfs25-dS/dS VLP (lanes 3 to 6). Cross reactivity of the anti-Pfs25 antibody with the dS scaffold protein was observed (Fig 1A, lanes 5 and 6). Therefore, Western blot analysis was repeated (shown in Fig A of the supporting information) with two different chimeric VLP preparations originating from two different *H. polymorpha* cell lines producing different amounts of two target proteins dS and Pfs25-dS. The blocking of the membrane was modified compared to the methodology described in the main manuscript. Instead of the Roti Block reagent (Carl Roth GmbH, Karlsruhe, Germany), 3 % (w/v) dry milk (in PBST) were applied as for the Pfs230-related Western blots. With this modified procedure we did not observe cross-reactivity of the anti-Pfs25 antibody with the dS in Western blot.

Lanes 2 and 4 in Fig A represent the actual Pfs25-dS/dS VLP preparation discussed in the main manuscript whereas lanes 1 and 3 represent a substantially lower concentrated Pfs25‑dS/dS VLP preparation originating from a different *H. polymorpha* cell line. In both VLP preparation the dS was detected by the anti-dS antibody (lanes 1 and 2). However, only for the preparation applied to lanes 2 and 4 the presence of the Pfs25-dS fusion protein was substantiated by the anti-Pfs25 antibody. Potentially, the Pfs25-dS fusion protein content in the sample applied to lanes 1 and 3 is below the detection limit. However, cross reactivity with the VLP scaffold protein dS was not observed for neither of the samples most likely due to the altered Western blot procedure compared to the analysis shown in the main manuscript (Fig 1 A).


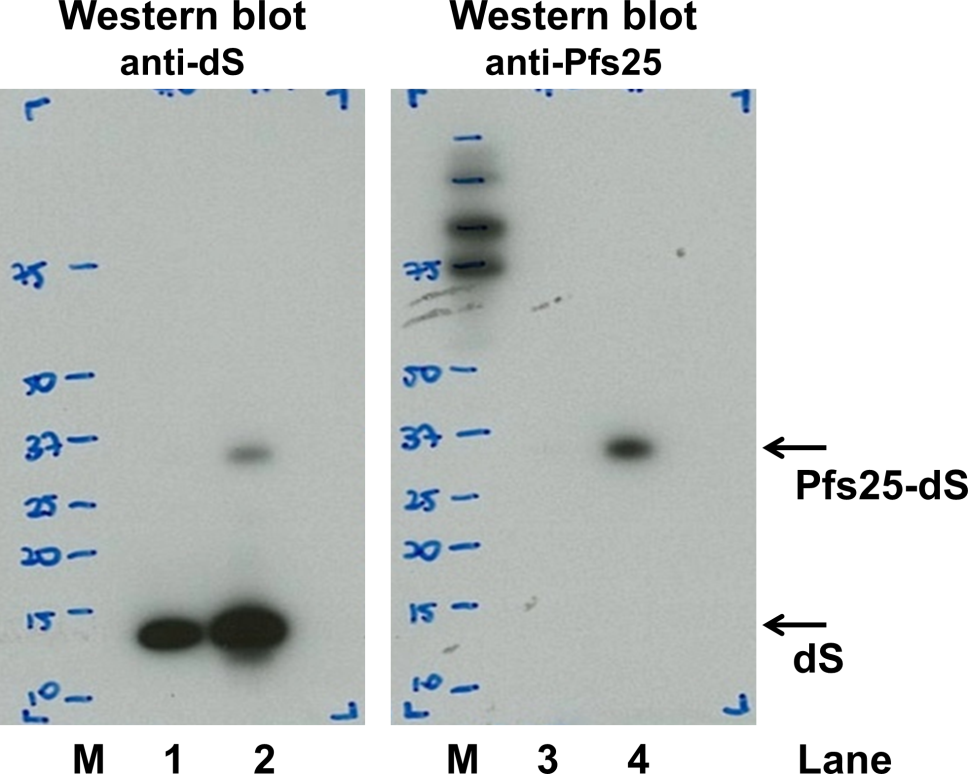


**Fig A**

**Repetition of Western blot analyses on chimeric Pfs25-dS/dS VLP.** Chimeric Pfs25-dS/dS VLP preparations obtained from two different H. polymorpha cell lines were applied. Lanes 2 and 4: the Pfs25-dS/dS VLP preparation discussed in the main manuscript. Lanes 1 and 3: A lower concentrated VLP preparation derived from a different cell line expressing lower levels of dS and Pfs25-dS. Left: Membrane probed with anti-dS 7C12 mAb. Right: Membrane probed with anti-Pfs25 mAb 32F81 and analyzed on the same membrane. M: molecular weight marker

**S1.2 Reduced product solubilization at elevated Pfs25-dS expression levels**

To overcome the low incorporation ratio of Pfs25-dS in the chimeric Pfs25-dS/dS VLP isolated from strain RK#097, additional strains co-expressing dS and Pfs25-dS were generated and screened for higher productive than strain RK#097 on the cell lysate level. One of them is the strain designated as DW#044. A side-by-side Western blot analysis of strain RK#097 and DW#044 is shown in Fig B. Cell pellets of the two strains were resuspended OD_600_ normalized in cell disruption buffer (25 mM Na-phosphate buffer, 2 mM EDTA, 0.5 % (w/v) Tween 20, pH 8.0). Cell disruption was carried out in 1.5 mL reaction tubes on a shaker (basic Vibrax shaker, IKA-Werke, Staufen, Germany) at maximal frequency for 30 min at 4 °C using glass beads (0.5–0.7 mm, Willy A. Bachofen, Nidderau-Heldenberg, Germany). One part of the resulting crude cell lysates was analyzed directly by anti-dS Western blot (lanes 1 and 4). The rest of the lysates was separated into soluble protein fraction (analyzed in lanes 3 and 6) and insoluble material (analyzed in lanes 2 and 5) by centrifugation (15 min, 13.000 *g,* 4 °C). The insoluble material was resuspended in distilled water volume-normalized to the volume of the centrifuged cell lysate prior to Western blot analysis. The comparison of lane 1 to lane 4 indicates higher productivity of the strain DW#044 compared to strain RK#097 regarding the fusion protein Pfs25-dS. However, in contrast to the material obtained from strain RK#097, the majority of the product proteins (dS and Pfs25-dS) produced by strain DW#044 was detected in the insoluble material (lane 5). Only a minority of the product was found in the soluble protein fraction (lane 6). The higher productivity on the cell lysate level (compare lanes 1 and 3) did not lead to higher product yields in the soluble protein fraction (compare lanes 3 and 6).


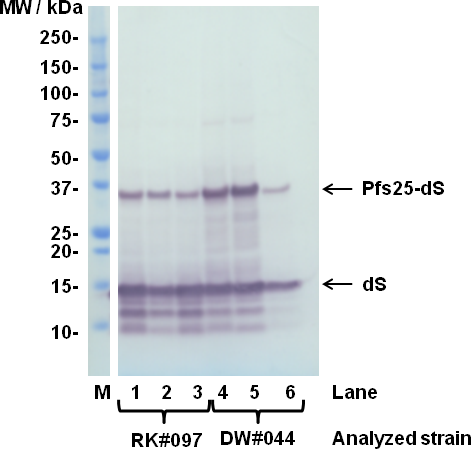


**Figure B**

**Side-by-side Western blot analysis of strains RK#097 and DW#044 co-producing the dS and Pfs25-dS.** Cell lysates (lanes 1 and 4), insoluble materials (lanes 2 and 5) and soluble protein fractions (lanes 3 and 6) obtained from equal amounts of cells were applied to the gel. The membrane was probed with anti-dS mAb 7C12. M: molecular weight marker.

This was analyzed in more detail by anti-dS Western blot analyses applying dilution series of the crude cell lysates and the soluble protein fractions (Fig C). The methodology of Western blot is only a semi-quantitative approach and the results have to be treated with caution. However, the decreased solubilization of the target proteins in case of the strain DW#044 (Fig C b) is obvious compared to the strain RK#097 (Fig C a). Based on analysis by densitometry approximately 54 % of the target proteins are solubilized in case of the strain RK#097 whereas only ~20 % of the target proteins were solubilized in case of the strain DW#044.

| 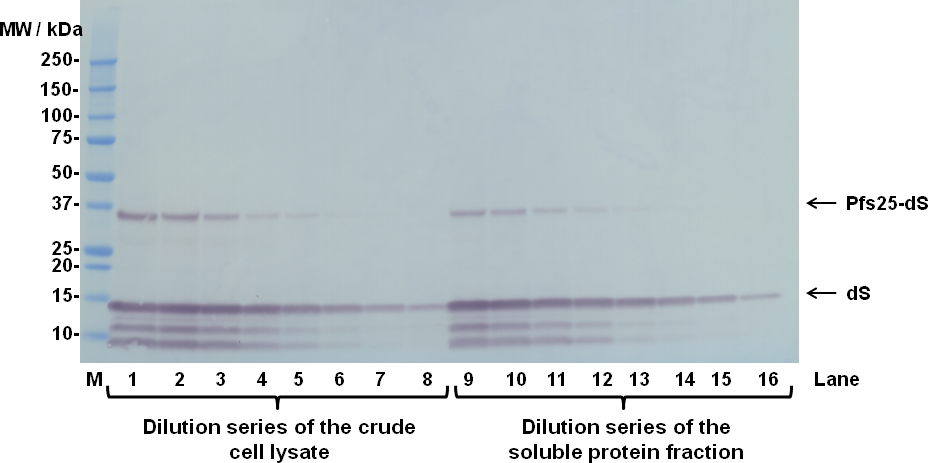 | **a** |
| --- | --- |
| 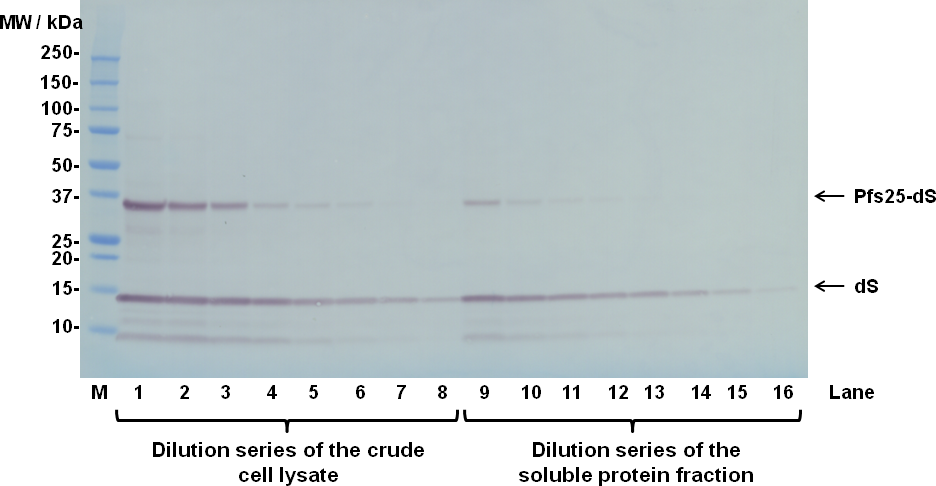 | **b** |

**Fig C**

**Western blot analyses of crude cell lysates and soluble protein fractions of strains RK#097 (a) and DW#044 (b).** The fractions were applied as dilution series (factor 2 steps). The membrane was probed with anti-dS mAb 7C12. M: molecular weight marker.

**Section S2: Additional data on the chimeric Pfs230c-dS/dS VLP**

**S2.1 Reactivity of different anti-Pfs230 immunoreagents with Pfs230c-dS/dS VLP in ELISA**

During the development of methodologies to analyze the Pfs230c-dS/dS VLP, different primary immunoreagents were tested. The mouse polyclonal antibody was found to be substantially more reactive than the 1B3 monoclonal antibody (Fig D).


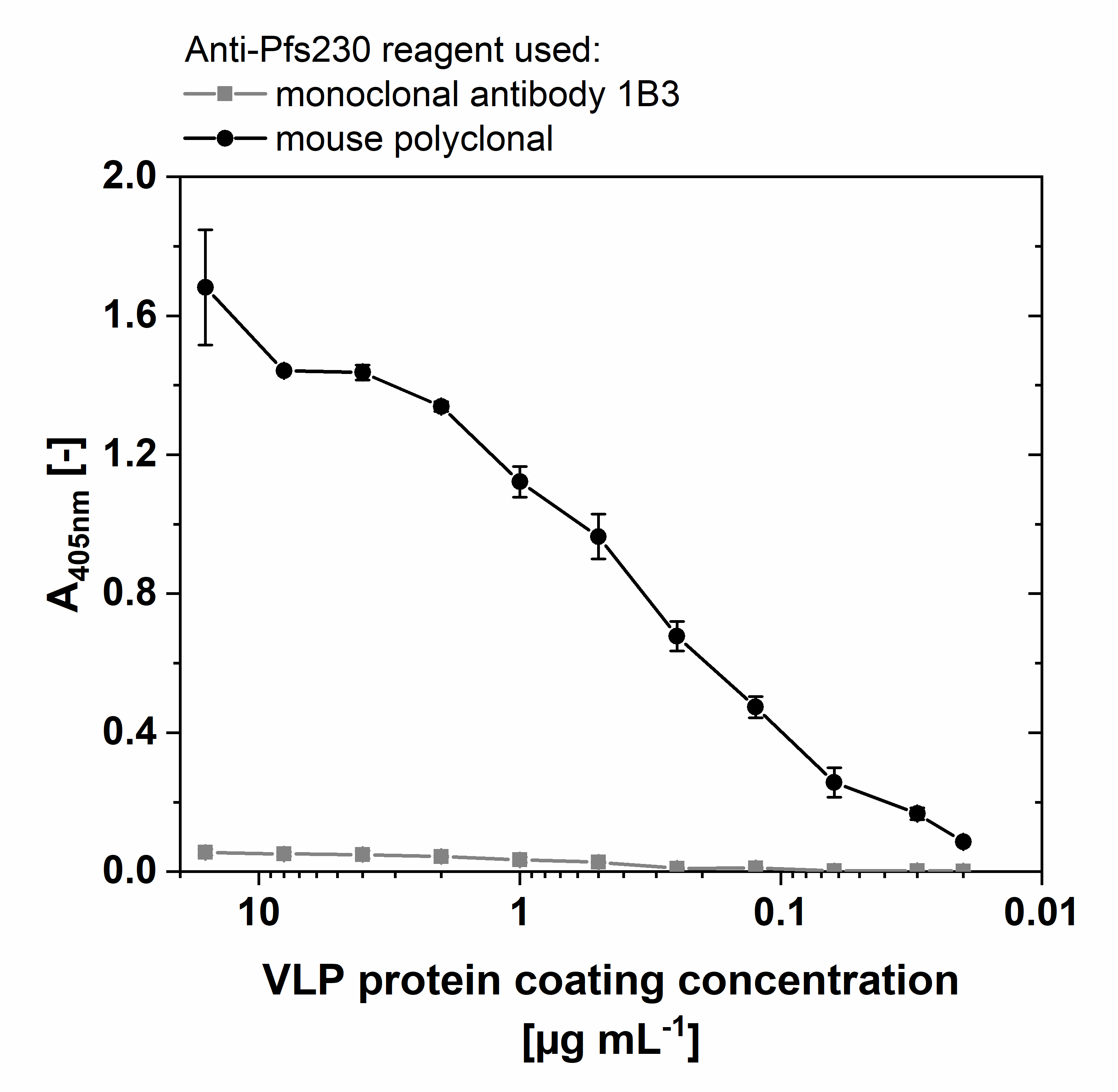


**Fig D**

**ELISA data on purified Pfs230c-dS/dS VLP derived from strain RK#114**. Titration of VLP coating concentration. Primary antibodies were applied as 10µg/mL for both the mouse polyclonal and monoclonal 1B3). Error bars indicate standard deviation based on triplicates.

**S2.2 Anti-HCP Western blot**

Anti-HCP Western blot was performed with the Pfs230c-dS/dS VLP preparation (Fig E, lane 1). The immunostaining of the membrane was performed as follows: Blocking with 1.5 % (w/v) powdered milk in PBS containing 0.05 % Tween 20 over-night at 4 °C. A polyclonal antiserum isolated from goats immunized with *H. polymorpha* HCP (Artes Biotechnology, Langenfeld, Germany/BioGenes, Berlin, Germany) was used as primary immunoreagent. The detection system was completed with a rabbit anti-goat IgG AP conjugate (BioRad, München, Germany) in combination with BCIP-NBT solution.

A subset of the bands detected in the Coomassie stained PAA gel (Fig E, lane 2) between the dS and the fusion protein was reactive with the polyclonal anti-HCP serum. Especially, the most prominent signals apart from the dS and the Pfs230c-dS in lane 2 could be identified as HCP in lane 1. Cross reactivity of the immunoreagents with the product-related proteins was not observed.


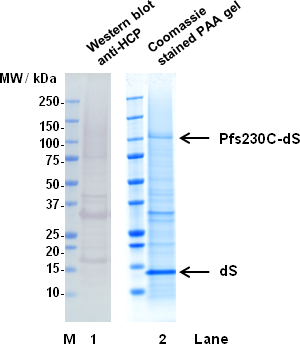


**Figure E**

**Anti-HCP Western blot analyses of purified Pfs230c-dS/dS VLP derived from strain RK#114.** The purified Pfs230c-dS/dS VLP preparation was analyzed by Western blot probed with anti-HCP serum (lane 1, 10 µg protein loaded) or Coomassie stained PAA gel (lane 2, 12 µg protein loaded). M: molecular weight marker.
